# The evolution of juvenile susceptibility to infectious disease

**DOI:** 10.1098/rspb.2018.0844

**Published:** 2018-06-20

**Authors:** Ben Ashby, Emily Bruns

**Affiliations:** 1 Department of Mathematical Sciences, University of Bath, Bath BA2 7AY, UK; 2 Department of Integrative Biology, University of California, Berkeley, CA, USA; 3 Department of Biology, University of Virginia, Charlottesville, VA, USA

**Keywords:** host–parasite, age structure, trade-off, susceptibility, development, eco-evolutionary theory

## Abstract

Infection prior to reproduction usually carries greater fitness costs for hosts than infection later in life, suggesting selection should tend to favour juvenile resistance. Yet, juveniles are generally more susceptible than adults across a wide spectrum of host taxa. While physiological constraints and a lack of prior exposure can explain some of this pattern, studies in plants and insects suggest that hosts may trade off juvenile susceptibility against other life-history traits. However, it is unclear precisely how trade-offs shape the evolution of juvenile susceptibility. Here, we theoretically explore the evolution of juvenile susceptibility subject to trade-offs with maturation or reproduction, which could realistically occur due to resource allocation during development (e.g. prioritizing growth over immune defence). We show how host lifespan, the probability of maturation (i.e. of reaching the adult stage) and transmission mode affect the results. Our key finding is that elevated juvenile susceptibility is expected to evolve over a wide range of conditions, but should be lowest when hosts have moderate lifespans and an intermediate probability of reaching the adult stage. Our results elucidate how interactions between trade-offs and the epidemiological-demographic structure of the population can lead to the evolution of elevated juvenile susceptibility.

## 1. Introduction

Hosts are likely to suffer higher fitness costs from disease if they are infected early in life, before they have had the opportunity to reproduce. Naively, then, we would predict that there should be strong selection for resistance early in life, and weaker selection for resistance later in life. However, empirical studies across a wide range of host taxa suggest that juveniles are almost always more susceptible to disease than adults [1–11]. Heightened juvenile susceptibility clearly has important epidemiological consequences. In humans, for instance, the spread of diseases such as measles and chicken pox is largely driven by children [12] and similar dynamics have been reported for many wildlife diseases [13,14]. Such examples highlight the epidemiological impact of juvenile susceptibility, yet we still lack a basic evolutionary understanding of precisely how and when juveniles are likely to be more susceptible than adults to infectious diseases.

In humans and other vertebrates this is primarily explained by the immunological naivety of juveniles who have yet to be exposed to (and hence acquire immunity to) many pathogens; still, there is growing evidence that prior exposure cannot fully explain patterns of age-specific susceptibility. For example, Baird [1] found that rates of malaria (*Plasmodium falciparum*) among Indonesian migrant families who moved from malaria-free to endemic conditions were higher for children than for their parents, and Kurtis *et al*. [11] showed that malaria parasitaemia decreases following the onset of puberty. Direct inoculation studies of bacterial and protozoan parasites in other vertebrates also support the general pattern of inherently higher juvenile susceptibility [2–4]. Higher juvenile susceptibility is especially evident in organisms that rely solely or primarily on innate forms of resistance. For example, the susceptibility of *Daphnia magna* to the bacterial pathogen *Pasteuria ramosa* appears to decrease with host age [8]. Inoculation studies with a wide variety of insects have also shown that disease susceptibility decreases with age [5–7]. In plants, greater susceptibility among juveniles has been documented in nearly every agriculturally important crop species [9].

Susceptibility at the juvenile stage has been widely assumed to be the result of strong physiological or developmental constraints on resistance [15,16], and perhaps as a result of this assumption, the evolutionary dynamics of juvenile susceptibility have yet to be thoroughly investigated. However, while developmental constraints undoubtedly contribute, they cannot completely explain the widespread pattern of juvenile susceptibility. For example, in plants, genetic variation for disease resistance at the seedling stage has been detected in a wide-range of wild species [17–19], and breeding has successfully led to marked improvements in seedling resistance of many crop plants [20,21], demonstrating that juvenile resistance is indeed physiologically possible. An alternative explanation may be that resistance at the juvenile stage trades off with increased growth or reproduction later in life, and these trade-offs are enough to maintain juvenile susceptibility. Trade-offs can occur, for example, due to resource allocation during development (e.g. prioritizing maturation or the growth of reproductive traits at the cost of weaker defence against infection during the juvenile stage) or pleiotropic effects (e.g. variable efficiency in nutrient uptake due to changes in cell surface receptors). Given that juveniles typically invest proportionately more resources in growth than adults, it is possible that hosts may temporarily divert resources away from immune defences during developmental stages in order to grow faster or larger, only investing in immune defences later in life when growth is less important. Resource allocation could therefore lead to a trade-off between juvenile susceptibility and the maturation rate (growing faster) or future reproductive output (growing larger). In plants, for example, where the trade-offs have been studied extensively, genes associated with juvenile resistance have been found to carry reductions as high as 9% in growth and reproduction [22–24].

While theoretical studies have investigated the effects of age-specific susceptibility on disease spread [25] and the evolution of resistance/susceptibility in non-age-structured populations [26–31], we are unaware of any general models that consider the evolution of juvenile susceptibility. Yet the underlying age structure and disease structure of the population may produce important epidemiological and demographic feedbacks that are hard to intuit without thoroughly analysing the dynamics. Disease prevalence will clearly be crucial in determining the realized cost of elevated juvenile susceptibility. The mode of transmission is, therefore, expected to play an important role, as pathogens with frequency-dependent as opposed to density-dependent transmission do not have extinction thresholds based on the size of the host population [32]. Here, we use a theoretical approach to understand what drives the evolution of juvenile susceptibility, assuming hosts trade-off juvenile susceptibility with maturation or reproduction during the adult stage. We show that juvenile susceptibility is generally high when hosts have short or long lifespans and low or high probabilities of reaching maturity, and is low in between, but the nature of the trade-off and mode of transmission can also affect the outcome.

## 2. Material and methods

We explore the evolution of elevated juvenile susceptibility, *β_J_*, to an infectious disease in a well-mixed, asexual host population where adult susceptibility, *β_A_*, is held constant. Fixing adult susceptibility allows us to focus on the conditions that lead to the evolution of higher juvenile susceptibility relative to the adult population. We assume that juveniles are always at least as susceptible to infection as adults, and that juvenile susceptibility is at most *d* times greater than adult susceptibility due to limitations of the pathogen (i.e. *β_A_* ≤ *β_J_* ≤ *dβ_A_*). We assume that elevated juvenile susceptibility arises due to a trade-off with either reproduction later in life, *a*(*β_J_*) = *a*_0_(1 + *z_a_*(*β_J_*)) (juveniles do not reproduce), or the maturation rate, *g*(*β_J_*) = *g*_0_(1 + *z_g_*(*β_J_*)), where *a*_0_ and *g*_0_ give the baseline reproduction and maturation rates (i.e. when juvenile and adult susceptibility are equal). For example, during development hosts may prioritize resources for growth rather than for defence against parasitism, which may lead to a shorter juvenile period or greater reproductive output as an adult at the cost of elevated susceptibility while juvenile. Since growth is more important during juvenile than during adult stages, we assume that the trade-off only occurs between juvenile susceptibility and maturation/adult reproduction. Thus, given a baseline level of susceptibility to infection (as expressed by adults), juveniles may temporarily divert resources away from immune defences, thus elevating susceptibility during development, but accelerating growth. Note that juvenile susceptibility will not be selected for *per se* because it is costly, but it may evolve due to trade-offs with beneficial traits such as higher maturation or reproduction rates. This is analogous to trade-offs typically employed in models for the evolution of virulence, where virulence (a costly trait) often evolves due to a trade-off with transmissibility (a beneficial trait) [33,34]. Here, the trade-off is defined by
2.1
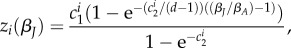
where *i* ∈ {*a*, *g*}, 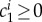 determines the strength of the relationship (the maximum reproduction rate is 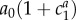 and the maximum maturation rate is 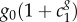, when *β_J_* = *dβ_A_*), and 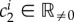 controls the shape of the trade-off. When 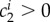 there are diminishing returns for elevating juvenile susceptibility (i.e. the costs accelerate), and when 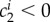 there are increasing returns (i.e. the costs decelerate). We restrict our analysis to a single trade-off at a time, setting 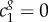 when 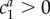, and vice versa. We assume that infected individuals have fecundity *f* relative to uninfected healthy individuals (0 ≤ *f* ≤ 1), and that they either die due to disease at an added rate *α* or recover without immunity at rate *γ* (recovery is generally fast relative to the maturation rate of the host, as would be expected for most acute infections). We set *S_J_* and *S_A_* (*I_J_* and *I_A_*) to be the densities of juvenile and adult individuals that are currently susceptible (infected), giving a total population density of *N* = *S_J_* + *S_A_* + *I_J_* + *I_A_*. Reproduction is subject to density-dependent competition (*q*) and there is no reproduction from juveniles. Hosts have an age-independent natural mortality rate of *b*; thus, in a disease-free population the average lifespan is 1/*b* and the baseline maturation probability (the probability of reaching the adult stage) is *g*_0_/(*b* + *g*_0_). The epidemiological dynamics are fully described by the following set of ordinary differential equations:
2.2*a*
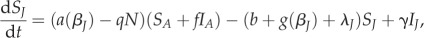

2.2*b*
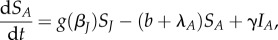

2.2*c*
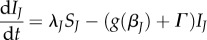

2.2*d*
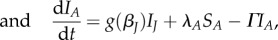
where *λ_J_* and *λ_A_* are the forces of infection experienced by juveniles and adults, respectively, and *Γ* = *b* + *α* + *γ* is the reciprocal of the infectious period. In the case of density-dependent transmission *λ_J_* = *β_J_*(*I_J_* + *I_A_*) and *λ_A_* = *β_A_*(*I_J_* + *I_A_*), whereas *λ_J_* = *β_J_*(*I_J_* + *I_A_*)/*N* and *λ_A_* = *β_A_*(*I_J_* + *I_A_*)/*N* when transmission is frequency-dependent.

Assuming mutations are rare (i.e. there is a separation of ecological and evolutionary timescales) and mutants are phenotypically similar to residents, the invasion fitness of a rare mutant is determined by the dynamics at the resident's ecological equilibrium. In the electronic supplementary material, we show that the invasion fitness of a rare mutant 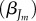 is sign equivalent to
2.3
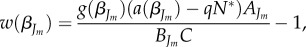
and the selection gradient is
2.4
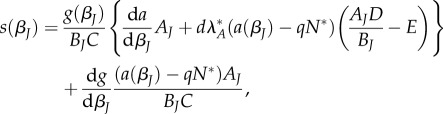
where 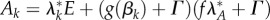, 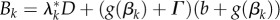, 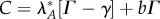, *D* = *g*(*β_k_*) + *Γ* − *γ* and *E* = 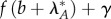 for *k* ∈ {*J*, *J_m_*,}. The trait will evolve in the direction of the selection gradient until a singular strategy, 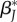, is reached at 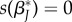, or until an extremum value of the trait is attained (i.e. *β_J_* = *β_A_* or *β_J_* = *dβ_A_*). The singular strategy is *evolutionarily stable* (ES; i.e. a local fitness maximum) if 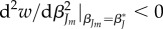 and is *convergence stable* (CS; i.e. locally attracting) if, for sufficently small *ε* > 0, *s*(*β_J_*) > 0 when 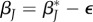 and *s*(*β_J_*) < 0 when 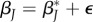. If the singular strategy is both ES and CS then it is a *continuously stable strategy* (CSS) [35]. We solve the dynamics numerically because the system is intractable to further algebraic analysis. We verify our results through simulations, which relax the adaptive dynamics assumptions of continuous traits and a complete separation of ecological and evolutionary time scales. Details of the simulations and the source code can be found in the electronic supplementary material.

## 3. Results

We explore the evolution of juvenile susceptibility by primarily focusing on the effects of host lifespan (1/*b*), the baseline probability of maturation (i.e. the probability of reaching the adult stage, *g*_0_/(*b* + *g*_0_)), the strength 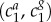 and shape 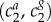 of the trade-offs, and the mode of transmission (density- or frequency-dependent). We begin by considering the case when transmission is density-dependent (*λ_J_* = *β_J_*(*I_J_* + *I_A_*) and *λ_A_* = *β_A_*(*I_J_* + *I_A_*)).

### (a) Qualitative outcomes

**Figure 1.**
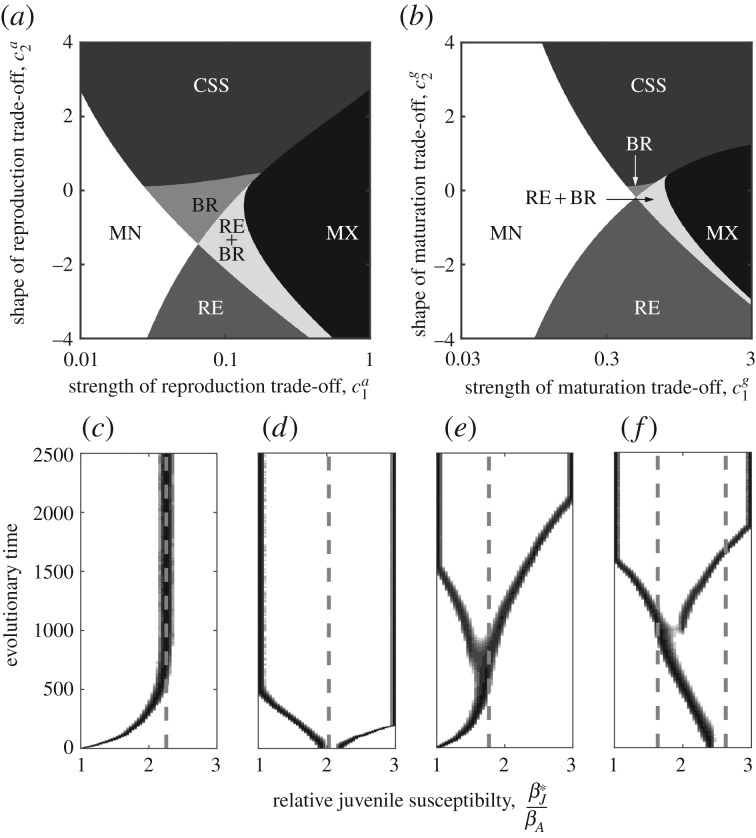
Qualitative outcomes for the evolution of juvenile susceptibility when the trade-off is against (*a*) adult fecundity and (*b*) maturation. (*a,b*) Trade-off spaces where the following outcomes occur: minimum susceptibility (i.e. *β_J_* = *β_A_*; MN); maximum susceptibility (i.e. *β_J_* = *dβ_A_*; MX); intermediate susceptibility (i.e. *β_A_* < *β_J_* < *dβ_A_*; CSS); repeller (RE); branching point (BR); repeller and a branching point (RE + BR). (*c*–*f*) Evolutionary simulations demonstrating some of these outcomes, with dashed lines indicating the singular strategies: (*c*) CSS; (*d*) RE; (*e*) BR; (*f*) RE + BR. Transmission is density-dependent. Fixed parameters: *a*_0_ = 2, *b* = 0.1, *d* = 3, *f* = 0.75, *g*_0_ = 0.25, *q* = 0.001, *α* = 0.5, *β_A_* = 2*q*/3, *γ* = 0.5.

The qualitative outcomes for the two types of trade-off are broadly similar to their strengths and shapes are varied (figure 1*a*,*b*). When either trade-off is relatively weak (small 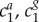) or strong (large 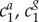), the host typically evolves to minimize 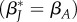 or maximize 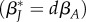 juvenile susceptibility, respectively (figure 1*a*,*b*). For more moderate relationships (intermediate 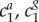) the qualitative outcome largely depends on the shape of the trade-off, with diminishing returns 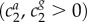 usually leading to a continuously stable strategy (CSS, 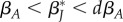; figure 1*c*), and increasing returns 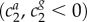 often giving rise to an evolutionary repeller (figure 1*d*). In the case of a repeller, the host evolves to either maximize or minimize juvenile susceptibility depending on the initial conditions. If the trade-off is of intermediate strength and is either decelerating 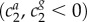 or very weakly accelerating 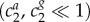, then evolutionary branching may occur, potentially dependent on the initial conditions (figure 1*e*,*f*). Evolutionary branching means that the population evolves towards a singular strategy, but then disruptive selection causes the population to diverge into two distinct branches. Typically, the two branches evolve to extreme trait values so that juveniles of one host type minimize susceptibility 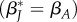 and juveniles of the other host type maximize susceptibility 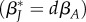.

### (b) Quantitative outcomes

We now consider how host lifespan and the baseline maturation probability quantitatively affect the evolution of juvenile susceptibility for the two trade-offs. We focus on the case when there are diminishing returns 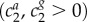 as this is when an optimal strategy may exist; note that for increasing returns 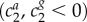 the host always minimizes or maximizes juvenile susceptibility (figure 1*a*,*b*).

#### (i) Reproduction rate trade-off

**Figure 2.**
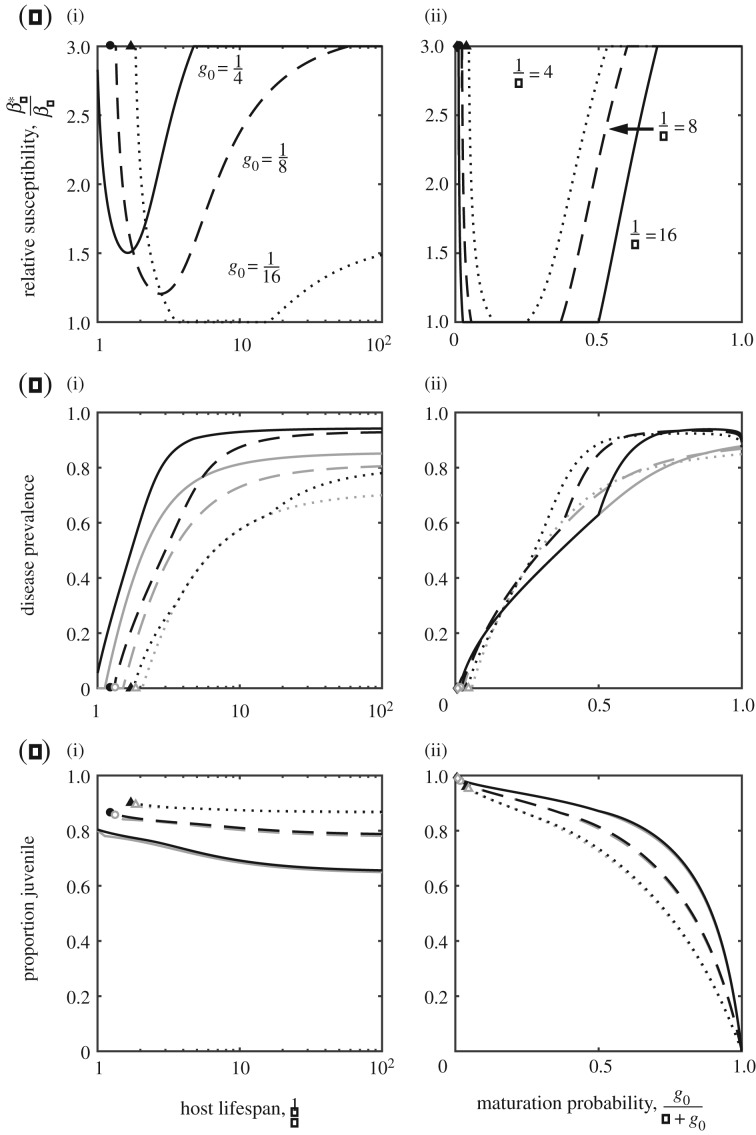
Evolution of juvenile susceptibility when the host experiences a diminishing trade-off with adult fecundity. (*a*(i),*b*(i),*c*(i)) Effects of host lifespan (1/*b*) for different maturation rates (*g*_0_). (*a*(ii),*b*(ii),*c*(ii)) Effects of the maturation probability (*g*_0_/(*b* + *g*_0_)) for different host lifespans. (*a*) Evolved level of juvenile susceptibility relative to the adult population. (*b*) Evolved (black) and initial (i.e. with *β_J_* = *β_A_*; grey) levels of disease prevalence. (*c*) Evolved (black) and initial (*β_J_* = *β_A_*; grey) proportion of the population that is juvenile. The solid, dashed and dotted lines in rows (*b*) and (*c*) correspond to those in row (*a*). The filled (evolved) and unfilled (initial) symbols indicate points at which the lines terminate because the host population is no longer viable. Transmission is density-dependent and parameters are as described in figure 1 with *a*_0_ = 5, 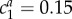, 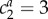 and *β_A_* = 2*q*.

When there is a trade-off between juvenile susceptibility and adult reproduction, selection for higher fecundity (hence higher juvenile susceptibility) is strongest when the host lifespan is either short or long and the probability of reaching the adult stage is low or high (figure 2*a*). This is because—all else being equal—disease prevalence decreases with shorter lifespans and when hosts are less likely to reach the adult stage (figure 2*b*), which increases selection for higher reproduction rates (and juvenile susceptibility) among hosts with these characteristics. Since disease is less common the costs of juvenile susceptibility are lower relative to the benefits of increased adult reproduction. Although disease prevalence is higher among hosts with longer lifespans and greater maturation probabilities, the proportion of hosts that are juvenile is lower (figure 2*c*), and so the relative costs of juvenile susceptibility are reduced. When hosts have intermediate lifespans or chances of reaching the adult stage, disease prevalence is likely to be at a moderate level and hosts spend a reasonable portion of their lives as juveniles, increasing the costs of juvenile susceptibility and thereby reducing selection for higher reproduction rates.

#### (ii) Maturation rate trade-off

**Figure 3.**
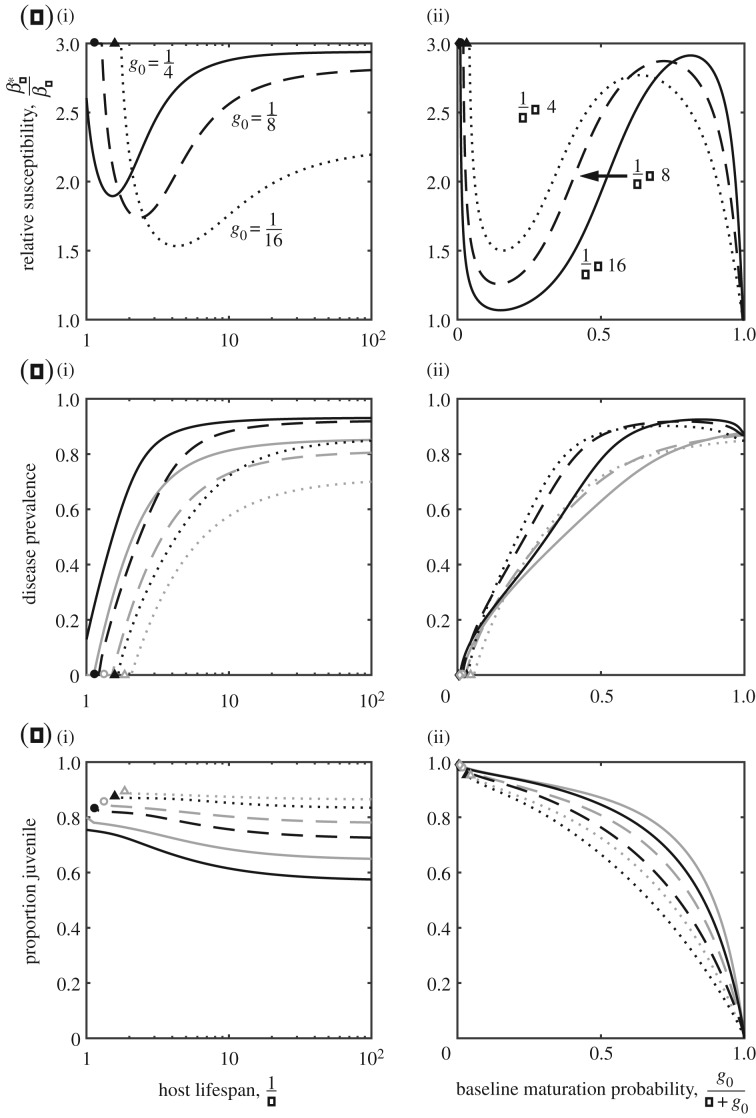
(*a*–*c*) Evolution of juvenile susceptibility when the host experiences a diminishing trade-off in terms of the maturation rate. Plots as described in figure 2. Transmission is density-dependent and parameters are as described in figure 1 with *a*_0_ = 5, 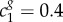, 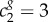 and *β_A_* = 2.

For the maturation rate trade-off, selection for shorter juvenile periods (hence higher juvenile susceptibility) is strongest when the host lifespan is short and is weakest for intermediate lifespans (figure 3*a*). As with the previous trade-off, this can be understood in terms of a balance between disease prevalence (figure 3*b*) and the age structure of the population (figure 3*c*). The relationship with the baseline maturation probability is slightly more complex (figure 3*a*(ii)). For the most part the relationship is similar to when the trade-off affects fecundity: high juvenile susceptibility evolves among hosts with low or fairly high baseline probabilities of reaching the adult stage, and is lower in between. The difference occurs among hosts with a very high baseline likelihood of attaining maturity 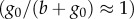, where evolution minimizes rather than maximizes juvenile susceptibility. This is because maturity is almost certain and so there is little advantage in increasing the probability of maturity any further. Hence, selection favours minimizing juvenile susceptibility rather than increasing an already fast maturation rate.

### (c) Frequency-dependent transmission

Finally, we consider the case when transmission is frequency-dependent (*λ_J_* = *β_J_*(*I_J_* + *I_A_*)/*N* and *λ_A_* = *β_A_*(*I_J_* + *I_A_*)/*N*). To ensure a fair comparison with density-dependent transmission, we calibrate *β_A_* so that the initial age structure and disease structure of the population matches the corresponding case when transmission is density-dependent. In other words, for a given set of parameters we calculate the initial equilibrium of the population when transmission is density-dependent (i.e. with *β_A_* = *β_J_*) and then we calibrate *β_A_* to generate the same age structure and disease structure for frequency-dependent transmission (provided the host is not driven extinct).

**Figure 4.**
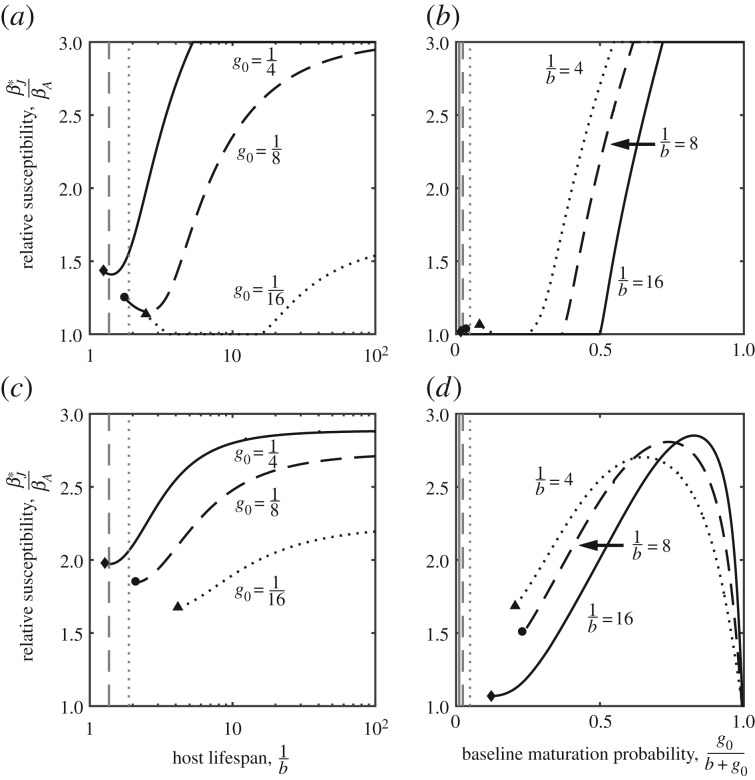
Evolution of juvenile susceptibility when transmission is frequency-dependent and the host experiences a diminishing trade-off in terms of the (*a,b*) reproduction rate or (*c,d*) maturation rate. Filled symbols indicate points at which evolved host population is driven extinct by the disease, and the vertical grey lines show the point at which the host population is initially viable (i.e. when *β_J_* = *β_A_*). The gap between the black and grey lines indicates where the host exhibits evolutionary suicide. Parameters as described in figure 1 with *a*_0_ = 5, 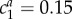 (in *a,b*), 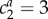, 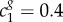 (in *c,d*), 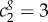, and *β_A_* adjusted so that the age structure and disease structure of the population matches that when transmission is density-dependent.

We find that the qualitative outcomes are broadly similar to those described for density-dependent transmission (figure 1; electronic supplementary material, S1), with the exception that for the maturation rate trade-off the branching region disappears, and instead a repeller and continuously stable strategy (RE + CSS) exist when the returns are weakly diminishing (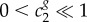) and are of intermediate magnitude 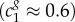. We again focus on the quantitative outcomes when the host experiences diminishing returns 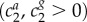, because hosts always evolve to minimize or maximize juvenile susceptibility when there are increasing returns 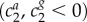. For moderate to long host lifespans and moderate to high baseline probabilities of maturation, the results closely match those for density-dependent transmission for both trade-offs (figure 4). The key difference occurs when hosts have short lifespans or low chances of reaching the adult stage, where the pathogen may be able to drive the host extinct, which is not possible when transmission is density-dependent [32]. In fact, selection for increased fecundity or a faster maturation rate, and as a result, juvenile susceptibility, can lead to evolutionary suicide by the host. This is shown in figure 4, where the threshold below which the population goes extinct (grey lines) is lower for unevolved populations than for evolved populations (black lines).

## 4. Discussion

Using a simple model, we theoretically explored the evolution of juvenile susceptibility due to a trade-off with maturation or reproduction as an adult. Our key result is that such trade-offs can lead to the evolution of elevated juvenile susceptibility over a wide range of conditions, but this is typically strongest for hosts with short or long lifespans and low or high probabilities of reaching the adult stage. This result can be understood in terms of a balance between the costs of disease prevalence, which tends to increase with host lifespan and the likelihood of maturation, and the benefits of reaching adulthood as host lifespan or the probability of maturation increases. However, there are some exceptions to our main result, for example, when the trade-off involves the maturation rate and the probability of maturation is very high (strong selection against juvenile susceptibility; figures 3*a*(ii) and 4*d*), or when transmission is frequency-dependent and either the host lifespan is short or the maturation probability is low (host evolutionary suicide; figure 4). In addition, the qualitative nature of the outcome depends on the shape and strength of any underlying trade-off (figure 1; electronic supplementary material, S1), with diversification occurring under certain conditions due to disruptive selection (figure 1*e*,*f*).

The fact that juvenile susceptibility is lowest among hosts with intermediate lifespans contrasts with results from non-age-structured models, where innate susceptibility is typically predicted to decrease with lifespan [26–31] (although see exceptions in [28] and [31]). In both types of model, disease prevalence increases with host lifespan, which makes susceptibility more costly. In the absence of age structuring, long-lived hosts tend to evolve lower innate susceptibility than short-lived hosts because they have a higher risk of infection. When the population is age-structured, however, the probability of successful maturation plays a crucial role, as hosts only reproduce during the adult stage and susceptibility may be age-dependent. All else being equal, long-lived hosts spend a smaller proportion of their life as juveniles, which is typically sufficient to offset the costs of juvenile susceptibility even though disease prevalence is high. Given that disease-prevalence is low among short-lived hosts, we therefore predict that juvenile susceptibility should be lowest for intermediate host lifespans and disease prevalence. This pattern is reminiscent of other host traits (such as recovery rate [36], mate choice [37] and sexual reproduction [38]) that are predicted to peak at intermediate disease prevalence.

We show that the type of trade-off, mode of transmission and probability of reaching the adult stage all impact the evolution of juvenile susceptibility. When the trade-off affects maturation rather than reproduction, juvenile susceptibility is minimized rather than maximized for hosts with very high chances of reaching the adult stage. Since maturity is almost certain, there is no advantage in shortening an already brief juvenile period (hence susceptibiitly remains low), but there is a strong benefit if hosts can increase their reproduction rate. We also found that the transmission mode plays an important role, potentially leading to evolutionary suicide in short-lived hosts. This occurs because there is no extinction threshold for the disease when transmission is frequency-dependent, and as a result even though disease is initially rare in short-lived hosts, selection for higher reproduction or maturation rates leads to greater juvenile susceptibility, which in turn increases disease prevalence to the point where the host is driven extinct. These dynamics are not possible when transmission is density-dependent because the pathogen is always driven extinct before the host [32]. Our model therefore predicts that in cases where disease transmission is frequency-dependent—for example, vector-borne diseases—hosts with shorter lifespans may be more at risk of evolutionary suicide due to trade-offs with juvenile susceptibility than hosts with longer lifespans. Sexual transmission is also typically thought to be frequency-dependent, but because juveniles are much less likely or unable to engage in sexual contact, sexually transmitted infections are unlikely to be important in the evolution of juvenile susceptibility.

A central assumption in the model was that hosts may trade off juvenile susceptibility against a faster maturation rate or greater reproduction during the adult stage. This is a reasonable assumption because there is: (1) a general precedent for trade-offs occurring between host susceptibility and growth or reproduction [39,40]; (2) indirect evidence of trade-offs involving juvenile susceptibility because juvenile resistance is often physiologically possible but does not evolve (e.g. variation in juvenile susceptibility among wild plants [17–19] and artificial selection for seedling resistance [20,21]); (3) direct evidence of reductions in growth and reproduction associated with genes conferring juvenile resistance in plants [22–24]; and (4) a realistic mechanism which could drive such trade-offs (resource allocation during development). Trade-offs involving juvenile susceptibility have been well documented in plants [22–24]. We have less information about the costs of age-specific resistance in animals, but costs of general innate [41] and induced immunity [42] have also been demonstrated. Future empirical studies need to focus more heavily on animal hosts to determine the nature of any trade-offs involving juvenile susceptibility.

Our model shows that even a moderate accelerating trade-off between juvenile susceptibility and adult reproduction or maturation can maintain juvenile susceptibility, without invoking physiological constraints [15,16]. Our model shows that, as in previous studies, the shape of the underlying trade-off is crucial in determining the qualitative outcome, with accelerating costs generally leading to a single continuously stable strategy (CSS) and decelerating or nearly linear costs necessary for repellers or evolutionary branching [27]. The shape and magnitude of any underlying trade-off will depend on the nature of the mechanism of resistance/susceptibility that is under selection. When evolutionary branching occurs in our model, hosts with elevated juvenile susceptibility are able to coexist with hosts that show no variation in juvenile–adult susceptibility due to the associated trade-off. Trade-offs may therefore explain observed variation in juvenile susceptibility, for example, among *Drosophila* [43] and wild plant species [17–19]. Interestingly, in certain regions of the parameter space it is possible for founder effects to determine whether the population evolves to be monomorphic or dimorphic (see [44] for similar dynamics). Two other assumptions of our model are that while juveniles and adults may differ in their susceptibility they remain equally infectious, and that juveniles and adults mix randomly. If juveniles and adults differ in infectiousness or mixing patterns, then this is likely to affect both the costs and benefits of juvenile susceptibility through changes in disease prevalence and the risk of infection for each life stage. Allowing for infectiousness and mixing patterns to differ would be an interesting extension to the current model, which may yield some interesting insights into more realistic populations. Still, all else being equal, longer lifespans and higher probabilities of reaching the adult stage will generally increase disease prevalence and reduce the relative duration of the juvenile stage, and so the overall patterns of our main results are likely to be broadly similar under these conditions.

These results provide an evolutionary explanation for a growing body of empirical evidence which shows that while juvenile resistance is physiologically possible, hosts may retain high levels of juvenile susceptibility [17–19,43], which cannot be entirely explained due to physiological constraints [15,16] or immunological naivety [1,2,4]. For example, in wild carnations (*Dianthus pavonius*), significant genetic variation for susceptibility to a sterilising disease (anther-smut) has been found at the seedling stage, yet demographic studies of a heavily diseased population have shown that juveniles maintain high levels of seedling susceptibility (10-fold that of adults) and account for the majority of transmissions [10].

Our results carry implications for understanding broad patterns in disease ecology, as juvenile susceptibility plays a critical role in the dynamics of many human [12], wildlife [13,14] and plant diseases [10]. Our model consistently predicts that longer-lived hosts are more likely to evolve elevated juvenile susceptibility, providing a window of opportunity for increased disease spread. Indeed, we show that the evolution of juvenile susceptibility in long-lived hosts results in an increase in disease prevalence. This could mean that juveniles play a more central role in disease transmission in long-lived hosts compared to shorter-lived hosts. Large-scale comparative studies or meta-analyses of disease transmission patterns across host lifespan would provide a critical test of this theory.

## Supplementary Material

Supplementary material

## Supplementary Material

Simulation source code

## Data accessibility

Simulation code is available in the electronic supplementary material.
