# Supplementary material for: The evolution of juvenile susceptibility to infectious disease
Source: Proc Biol Sci. 2018 Jun 20;285(1881):20180844. doi: 10.1098/rspb.2018.0844 (PMC6030539; doi:10.1098/rspb.2018.0844)
Supplement: Supplementary material [file rspb20180844supp1.pdf]

Supplementary material: The evolution of juvenile susceptibility to infectious disease

Ben Ashby[1,2,*] & Emily Bruns[3]

1. Department of Mathematical Sciences, University of Bath, Bath, BA2 7AY, UK.
2. Department of Integrative Biology, University of California, Berkeley, Berkeley, CA, USA.
3. Department of Biology, University of Virginia, Charlottesville, VA, USA.

*Corresponding author: benashbyevo@gmail.com

S1. EPIDEMIOLOGICAL DYNAMICS

The disease-free equilibrium occurs at

$$S_J^* = \frac{bS_A^*}{g(\beta_J)} \tag{S1a}$$

$$S_A^* = \frac{g(\beta_J)\big(a(\beta_J) - b\big) - b^2}{q\left(b + g(\beta_J)\right)} \tag{S1b}$$

which is stable provided $g(\beta_J)\big(a(\beta_J) - b\big) > b^2$ and the basic reproductive ratio, R_0, of the disease is less than 1. The disease can invade provided the $R_0 > 1$, with

$$R_0 = \frac{S_A^*\left(b\beta_J + \beta_A g(\beta_J)\right)}{g(\beta_J)\Gamma} \tag{S2}$$

when transmission is density-dependent, and

$$R_0 = \frac{b\beta_J + \beta_A g(\beta_J)}{\Gamma\left(b + g(\beta_J)\right)} \tag{S3}$$

when transmission is frequency-dependent. There is no general expression for the endemic equilibrium, but extensive simulations suggest that it is likely to always be asymptotically stable provided $g(\beta_J)(a(\beta_J) - b) > b^2$ and $R_0 > 1$ (although the disease can potentially drive the host extinct when transmission is frequency-dependent).

S2. Derivation of host fitness and selection gradient

We derive the invasion fitness of a rare mutant using the next-generation method (Hurford *et al.* 2010). The Jacobian of the rare mutant dynamics is:

$$J = \begin{pmatrix} -b - g(\beta_{J_m}) - \lambda_{J_m}^* & a(\beta_{J_m}) - qN^* & \gamma & f\big(a(\beta_{J_m}) - qN^*\big) \\ g(\beta_{J_m}) & -b - \lambda_A^* & 0 & \gamma \\ \lambda_{J_m}^* & 0 & -g(\beta_{J_m}) - \Gamma & 0 \\ 0 & \lambda_A^* & g(\beta_{J_m}) & -\Gamma \end{pmatrix} \quad (S4)$$

which can be split into components F and V such that $V = F - J$, where:

$$F = \begin{pmatrix} 0 & a(\beta_{J_m}) - qN^* & 0 & f\big(a(\beta_{J_m}) - qN^*\big) \\ 0 & 0 & 0 & 0 \\ 0 & 0 & 0 & 0 \\ 0 & 0 & 0 & 0 \end{pmatrix} \quad (S5)$$

$$V = \begin{pmatrix} b + g(\beta_{J_m}) + \lambda_{J_m}^* & 0 & -\gamma & 0 \\ -g(\beta_{J_m}) & b + \lambda_A^* & 0 & -\gamma \\ -\lambda_{J_m}^* & 0 & g(\beta_{J_m}) + \Gamma & 0 \\ 0 & -\lambda_A^* & -g(\beta_{J_m}) & \Gamma \end{pmatrix} \quad (S6)$$

The next generation matrix is then $N_G = FV^{-1}$ and the invasion fitness of the rare mutant, $w\big(\beta_{J_m}\big)$, is sign equivalent to the largest eigenvalue of N_G minus 1:

$$w\big(\beta_{J_m}\big) = \frac{g(\beta_{J_m})\big(a(\beta_{J_m}) - qN^*\big)A_{J_m}}{B_{J_m}C} - 1 \quad (S7)$$

where the following terms are used to simplify the notation:

$$A_{J_m} = \lambda_{J_m}^* E + \big(g(\beta_{J_m}) + \Gamma\big)(f\lambda_A^* + \Gamma) \quad \text{(S8a)}$$

$$B_{J_m} = \lambda_{J_m}^* D + \big(g(\beta_{J_m}) + \Gamma\big)\big(b + g(\beta_{J_m})\big) \quad \text{(S8b)}$$

$$C = \lambda_A^*[\Gamma - \gamma] + b\Gamma \quad \text{(S8c)}$$

$$D = g(\beta_{J_m}) + \Gamma - \gamma \quad (S8d)$$

$$E = f(b + \lambda_A^*) + \gamma \quad (S8e)$$

The selection gradient, $s(\beta_J) = \left.\frac{dw}{d\beta_{J_m}}\right|_{\beta_{J_m}=\beta_J}$, is therefore:

$$s(\beta_J) = \frac{g(\beta_J)}{B_J C}\left\{\frac{da}{d\beta_J}A_J + d\lambda_A^*\big(a(\beta_J) - qN^*\big)\left(\frac{A_J D}{B_J} - E\right)\right\} + \frac{dg}{d\beta_J}\frac{\big(a(\beta_J) - qN^*\big)A_J}{B_J C} \quad (S9)$$

and β_J^* is a *singular strategy* if $(\beta_J^*) = 0$. A singular strategy is *evolutionarily stable* if:

$$ES(\beta_J^*) = \left.\frac{d^2 w}{d\beta_{J_m}^2}\right|_{\beta_{J_m}=\beta_J^*} < 0 \quad (S10)$$

S3. EVOLUTIONARY SIMULATIONS

The above method assumes that mutations are rare (a complete separation of ecological and evolutionary timescales) that mutations have very small phenotypic effects. We relax these assumptions in our simulations, which allow mutations of larger size to occur when the system has yet to reach its dynamical attractor (source code available in the online *Supplementary Material*). Starting with a single resident trait, β_J, we solve the ODE system for a given time period $[0, T]$ ($T = 500$), then introduce a mutant, $\beta_{J_m} = \beta_J \pm \epsilon_1$ (mutation size $\epsilon_1 = (d-1)/50$), at low frequency. We then rerun the ODE solver over the period $[T, 2T]$ and remove any strains that have fallen below a frequency of $\epsilon_2 = 10^{-5}$. If more than one trait is still present in the population, then the next

mutant is chosen based on a weighted probability of the trait frequencies. The process is repeated for n = 5000 iterations.

REFERENCES

Hurford, A., Cownden, D. & Day, T. (2010). Next-generation tools for evolutionary invasion analyses. *J. R. Soc. Interface* 7:561–571.

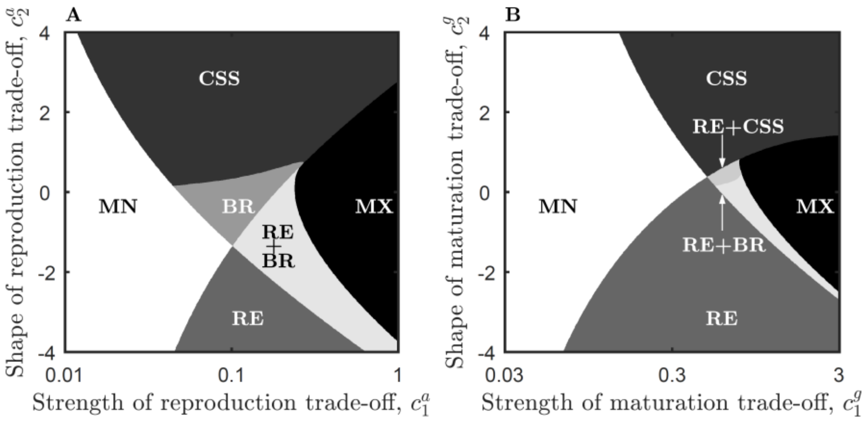

Figure S1 - Qualitative outcomes for the evolution of elevated juvenile susceptibility when disease transmission is frequency-dependent. In (A) there is a trade-off between juvenile susceptibility and adult fecundity (equation 1a) and in (B) the trade-off occurs with the maturation rate. The outcomes are as described in Fig. 1, with the addition of a repeller and a continuously stable strategy (RE+CSS). β_A is calibrated to match the initial age- and disease-structure of the population when transmission is density-dependent. Remaining parameters as described in Fig. 1.
